# A Method for Determining the Fracture Toughness of Shotcrete Materials Subjected to Freeze–Thaw Cycles

**DOI:** 10.3390/ma18010171

**Published:** 2025-01-03

**Authors:** Xiangyu Han, Qilong Zhao, Bin Jia, Jinqiao Liu, Qionglin Li, Quan Zhang

**Affiliations:** 1 School of Civil Engineering and Architecture, Southwest University of Science and Technology, Mianyang 621010, China; 2 China Railway Construction Kunlun Metro Investment and Construction Management Co., Ltd., Chengdu 610031, China; 3 School of Civil Engineering, Southwest Jiaotong University, Chengdu 610031, China

**Keywords:** shotcrete, fracture toughness, freeze–thaw cycles, microstructure

## Abstract

Defects can be introduced into shotcrete materials after a few freeze–thaw cycles, which has a significant influence on the fracture performance of shotcrete. In this study, a series of shotcrete specimens with varying sizes, geometries, and initial crack lengths were prepared to investigate the fracture properties of notched shotcrete under freeze–thaw conditions. Considering the effects of specimen boundaries and material microstructure, a linear closed-form solution was proposed to determine the fracture toughness of frost-damaged shotcrete. The fracture toughness was found to be a reliable material constant, independent of specimen geometry variations. Results from three-point bending (3PB) tests show that freeze–thaw cycles severely weaken the fracture toughness of shotcrete, which is consistent with CT scan images of the damaged microstructure of the shotcrete specimens. Moreover, specimens with longer initial notches exhibited more severe freeze–thaw damage, which should be carefully considered in practical engineering assessments. These findings highlight the critical importance of considering freeze–thaw effects and notch length when evaluating the durability of shotcrete in cold region applications.

## 1. Introduction

Due to their excellent workability and mechanical performance, shotcrete materials have been applied as support measures in various engineering, such as tunnel engineering, slope engineering, dam engineering, etc. [1,2,3]. However, weak points can be introduced into shotcrete materials during the spraying procedure. This means that shotcrete is a material inherently characterized by defects [4]. When subjected to freeze–thaw cycles, shotcrete is prone to experiencing deterioration at these weak points. Consequently, the fracture resistance of shotcrete tends to weaken accordingly [5], making fractures one of the most common failure types in deteriorated shotcrete.

In recent decades, researchers have focused their attention on the effect of freeze–thaw cycles on properties of concrete materials in cold regions. Typically, water-saturated concrete in cold environments experiences two types of stress-induced damage: (1) swelling pressure due to the freezing process within the capillary pores of the concrete, and (2) osmotic pressure generated by permeation along the edges of gel pores [6,7,8]. During freeze–thaw cycles, the pore structures of concrete repeatedly endure expansion and osmotic forces, resulting in continuous damage accumulation. Consequently, initially minute voids evolve into clearly visible fissures, ultimately leading to a reduction in concrete strength. Huang et al. studied the influence of freeze–thaw cycles on the cubic compressive strength of concrete, and a 38.3% strength loss was witnessed after 300 cycles [9]. Li et al. found that the elastic modulus experienced a 75% decrease after 240 cycles of the freeze–thaw process [10]. Islam et al. investigated the stiffness degradation behavior of asphalt concrete under long-term freeze–thaw cycles, where a 17% decrease in stiffness was observed in the first 20 cycles [11]. Chen et al. studied microstructural damage in shotcrete under a freeze–thaw environment and proposed changing the rule of axial compressive strength of damaged shotcrete [12]. It can be concluded that the properties of concrete materials are severely altered by freeze–thaw environment.

Concrete-fracture-related studies are crucial for understanding suitable applications, with the study of fracture models being a perennial research hotspot. Hillerborg proposed a fictitious crack model to describe the stress distribution along the crack tip, and this model has been widely applied in the numerical simulation of concrete fracture [13,14]. Bazant compared the fracture of concrete specimens with different sizes, three different equations were used to calculate the fracture properties for different specimen types, and the size effect law is established to illustrate the size effect phenomenon of fracture properties [15,16,17]. Xu and Reinhardt considered the fracture of concrete to consist of two stages (initial cracking and unstable fracture); they proposed double-K model to calculate the fracture toughness values above two critical stages [18,19,20,21]. However, these models have rarely been utilized to determine the fracture properties of shotcrete under freeze–thaw conditions. A concise method for determining the fracture properties of shotcrete under freeze–thaw conditions is urgently needed, particularly for practical applications in cold region engineering projects, such as tunnel linings, dams, and other infrastructure exposed to extreme weather. Engineers must assess the material’s durability quickly and accurately to ensure long-term structural integrity. Traditional fracture testing methods can be time-consuming and resource-intensive, making them less suitable for rapid field assessments. Therefore, a practical and efficient evaluation technique would enable engineers to evaluate shotcrete’s fracture performance under freeze–thaw conditions more effectively, allowing for timely decision making in the design, maintenance, and repair of critical structures.

In present study, the method for calculating fracture properties of notched three-point bending (3-p-b) shotcrete specimens is proposed, the tests of various conditions are adopted to verify its reliability. The paper is organized as follows: Section 2 describes the preparation of shotcrete specimens and fracture tests, Section 3 outlines the process of establishing fracture calculation methods, Section 4 analyzes the applicability of proposed methods under various conditions and discusses the fracture characteristics of frost-damaged shotcrete, Section 5 presents conclusions.

## 2. Fracture Tests of Shotcrete Specimens After Freeze–Thaw

The shotcrete materials were prepared in the laboratory to ensure the consistency of the specimens. These materials comprised coarse aggregates (with a maximum size of 10 mm)—sand, cement, water, fly ash, a superplasticizer, and an accelerator—with a mass ratio of 808:875:456:182:29:4.85:33.95. The superplasticizer was employed to improve workability and enhance shotcrete compaction, while the accelerator was utilized to increase setting time and achieve significant early strength, crucial for supporting engineering applications. Detailed information on the superplasticizer and accelerator can be found in Table 1 and Table 2, respectively. The manufacturing process of shotcrete specimens involved the following steps: preparing the raw materials according to the designed ratio; mixing the aggregates, cement, water, and superplasticizer in a mixer; pumping the mixtures using a wet jet machine, and spraying the mixtures into molds. It is important to note that compressed air (0.45–0.60 MPa) was utilized to provide the spraying power, and the accelerator was added at the nozzle location to ensure uniform distribution.

**Figure 1:**
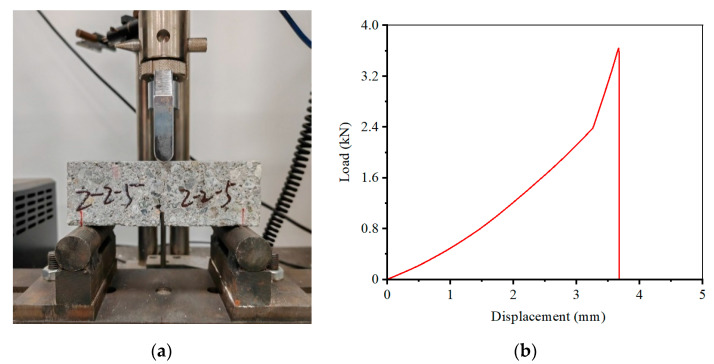
The three-point bending tests of freeze–thaw damaged shotcrete: (**a**) test process; (**b**) load–displacement curves.

After the shotcrete reached its initial setting time, the plates were cut into specific sizes, as detailed in Table 3. Through-thickness notches were also introduced at the center of the specimens. Subsequently, the specimens were cured in a water tank for 28 days. Following this immersion period, the specimens were transferred to a freeze–thaw testing machine. To mimic the extreme environmental conditions that shotcrete may encounter in engineering applications, the temperature range was set from −25 to 10 degrees Celsius, with each freeze–thaw cycle lasting 4.5 h. Importantly, all specimens were completely submerged in water to ensure the saturation of the shotcrete materials, including the notches which were filled with water. The freeze–thaw conditions for different groups are outlined in Table 3. Following exposure to freez–thaw damage, three-point bending fracture tests were performed on these shotcrete specimens, with a loading rate set at 0.1 mm/min, as shown in Figure 1. During testing, the fracture process was meticulously documented, the load–displacement curves were measured, and the average failure loads of different groups are presented in Table 3.

## 3. The Calculation Methods of Shotcrete Fracture Toughness

Fracture toughness is a fundamental parameter for characterizing the fracture behavior of concrete materials. Ideally, fracture toughness should be a material constant, independent of the geometrical size of specimens. Therefore, when calculating the fracture toughness of concrete materials, it is essential to carefully consider the influence of material microstructures and specimen size [22,23,24]. The Boundary Effect Model (BEM), proposed by Hu, emphasizes the significance of the distance between the crack tip and specimen boundaries in the fracture analysis of quasi-brittle materials [25,26,27,28,29]. Based on the BEM, calculation methods are established to determine the fracture properties of shotcrete specimens under freeze–thaw cycles. The key aspects of the calculating methods are outlined below.

(1) $$\sigma_{n}=\frac{f_{t}}{\sqrt{1+\frac{a_{e}}{a_{ch}^{*}}}}$$

(2) $$a_{e}={\left[\frac{{\left(1-\alpha \right)}^{2}\times Y(\alpha )}{1.12}\right]}^{2}a_{0}$$

(3) $$a_{ch}^{*}=0.25\times {\left(\frac{K_{IC}}{f_{t}}\right)}^{2}=3G$$

where *σ_n_* is the nominal stress of finite-sized specimens, *f*_*t*_ is tensile strength, and *a_e_* is the equivalent crack length. The characteristic crack length $a_{ch}^{*}$ is relevant to the microstructure of material and establishing a correlation between tensile strength (*f_t_*) and fracture toughness (*K_IC_*). G could be set as the half value of maximum aggregate size. *α* is the ratio of initial crack length (*a*_0_) and specimen height *W*, *Y(α)* is the geometry factor which determined by *α* and specimen geometry.

**Figure 2:**
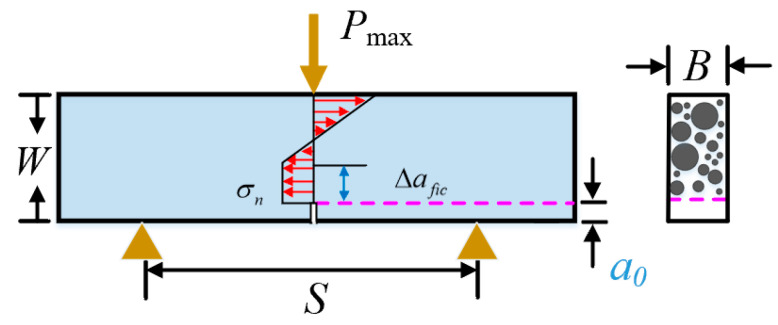
The illustration of three-point bending shotcrete specimens.

For three-point bending specimens, the stress distribution along unnotched ligament is assumed as shown in Figure 2 [30,31]. The relation between maximum failure load and nominal stress can be depicted as follows:

(4) $$\sigma_{n}=\frac{1.5\times \frac{S}{B}\times P_{\max}}{\left(W-a_{0})(W-a_{0}+2\Delta a_{fic}\right)}$$

Combining Equation (1) with Equation (4), the relationship between maximum failure load and fracture toughness can be established as Equation (5).

(5) $$K_{IC}=P_{\max}/B_{e}(W,a_{0},G)=P_{\max}\times \frac{3\cdot \sqrt{3G}\times (\frac{S}{B})\times \sqrt{1+\frac{a_{e}}{3G}}}{\left(W-a_{0})\times (W-a_{0}+\Delta a_{fic}\right)}$$

As shown in Equation (6), the fictitious crack length $∆a_{fic}$ can be described by the average aggregate size (*G*) and discrete number (*β_av_*), where the influence of specimen boundary, pore structure and aggregate is well considered. For laboratory-casted concrete specimens, for which size is limited, the value of *β_av_* could be set as 1.5 [32].

(6) $$\Delta a_{fic}=\beta_{av}\times G$$

The geometry factor *Y*(α) could be determined as follows:

(7) $$Y_{2.5}(\alpha )=\frac{1-2.5\alpha +4.49\alpha^{2}-3.98\alpha^{3}+1.33\alpha^{4}}{{\left(1-\alpha \right)}^{3/2}}\;(S/W\;=2.5)$$

(8) $$Y_{4.0}(\alpha )=\frac{1.99-\alpha (1-\alpha )(2.15-3.93\alpha +2.7\alpha^{2})}{\sqrt{\pi }(1+2\alpha ){\left(1-\alpha \right)}^{3/2}}\;(S/W\;=4.0)$$

(9) $$Y(\alpha )=\frac{S/W-2.5}{1.5}Y_{4.0}(\alpha )-\frac{S/W-4}{1.5}Y_{2.5}(\alpha )\;(S/W\;=2.0\sim 4.0)$$

The distribution of fracture properties has been proved to follow a normal distribution for concrete, hard rock, bamboo composites, fibrous composites, etc. [33,34,35,36] Hence, the normal distribution analysis method, Equation (10), is incorporated into Equation (5), the experimental scatters can be well described, as shown in Equation (11), where *μ* and *σ* are the mean and standard deviation values of calculated fracture toughness, respectively.

(10) $$f(x)=\frac{1}{\sqrt{2\pi }\sigma }e^{-\frac{{\left(x-\mu \right)}^{2}}{2\sigma^{2}}}$$

(11) $$P_{\max}=(\mu \pm \sigma )\times B_{e}(W,a_{0},G)$$

**Figure 3:**
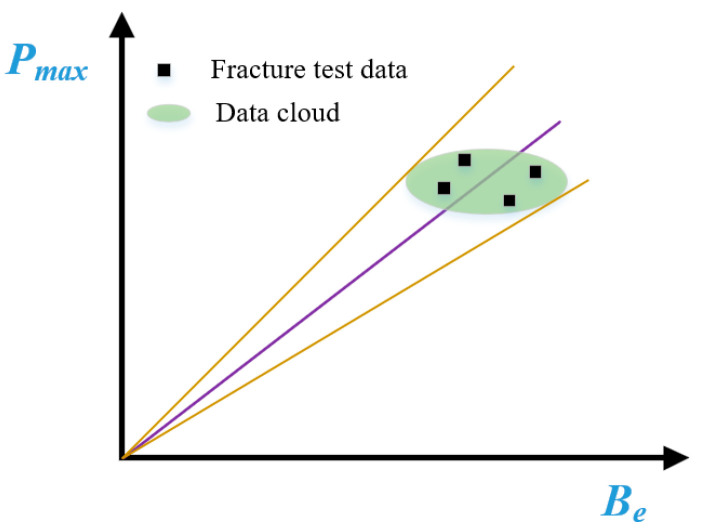
Determining the fracture toughness of shotcrete with scattered test results.

In this way, the methods for determining the fracture toughness of shotcrete materials are constructed as a linear line that crosses the origin, as shown in Figure 3. Once the 3-p-b tests of frost-damaged shotcrete specimens are conducted, the failure loads are easily obtained. *B_e_* (*W*, *a*_0_, *G*) is already determined for each specimen, and then the fracture toughness value can be calculated using the slope of the linear line, according to Equation (5). However, the scattered distribution has been widely accepted as the characteristics of concrete fracture test results. Hence, a data cloud (more than four specimens) is always needed to determine the distribution of shotcrete fracture toughness by virtue of Equation (10); then, the mean value can be considered as the real fracture toughness of shotcrete materials. Moreover, the standard deviation value could also help to establish the confidence interval, which is useful for model verification and fracture prediction.

## 4. Application of the Fracture Toughness Calculated Model

### 4.1. Calculation of Fracture Toughness Values of Frost-Damaged Shotcrete

**Figure 4:**
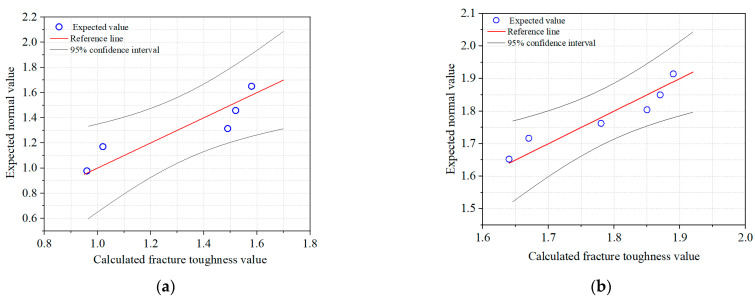
Distribution analysis of fracture toughness values with quantile–quantile plot: (**a**) Group A, (**b**) Group E.

After collecting the maximum failure loads of frost-damaged shotcrete specimens from various groups, the fracture toughness of each specimen could be easily calculated following the steps in Section 3. It should be noted that some specimens were broken during the freeze–thaw process. In particular, when the number of freeze–thaw cycles reached 100, the number of broken specimens began to increase. Therefore, only valid data were listed and calculated, as shown in Table 4. A huge variation can be witnessed among maximum failure loads, even for the specimens with identical sizes and geometries. Such scattered distributions always become an obstacle for determining the constant fracture properties of concrete materials. In fact, the dispersed distribution is a characteristic of the mechanical properties of concrete materials and is unavoidable. Especially for damaged shotcrete, a large amount of deviation is introduced during casting, freeze–thaw cycles, and fracture testing. Therefore, it is particularly important to determine the distribution characteristics of the fracture parameters of damaged shotcrete. In this section, the fracture toughness values in groups A and E are selected to analyze the distribution characteristics by using a quantile–quantile plot, as shown in Figure 4. It could be found that five data points in Group A and six data points in Group E all fell into the confidence interval region and were distributed around the reference lines. Namely, the fracture toughness values of both groups follow a normal distribution relatively well.

### 4.2. The Influence of Specimen Geometry

**Figure 5:**
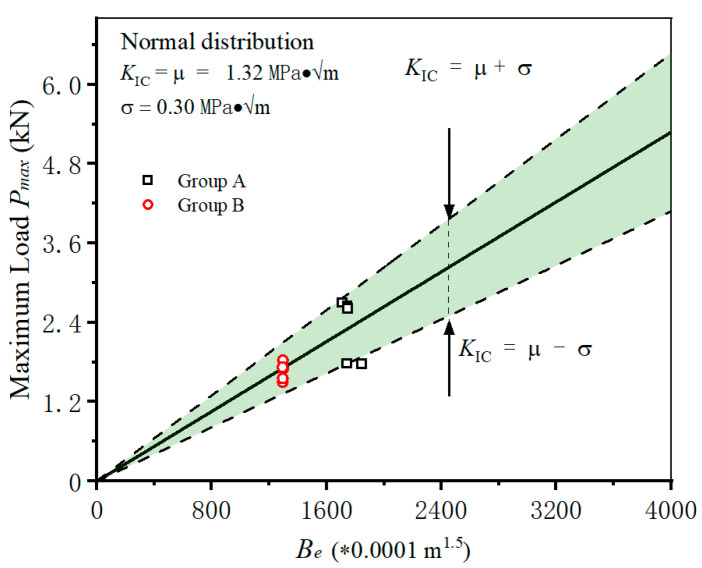
Predicting the fracture of specimens in B with determined fracture toughness from A.

In practical engineering, shotcrete is often cast into various geometries. However, a real fracture toughness should be determined as constant regardless of the variation in specimen geometry. Therefore, the shotcrete specimens with different geometries in Group A (*S/W* = 3) and Group B (*S/W* = 4) are adopted to study the influence of specimen geometries on calculating the fracture toughness of shotcrete. The fracture test results in Group A are firstly used to determine the fracture toughness of shotcrete materials, the normal distribution analysis is also introduced here. The distribution of fracture toughness yields a mean value of *μ* = 1.32 MPa·√m and standard deviation value of *σ* = 0.30 MPa·√m. Then, the triangle predictive area is constructed on the basis of Equation (11), and fracture toughness is determined, as shown in Figure 5. After that, the fracture test results of Group B are plotted using the same coordinates. It can be found that the scatters of B all fall into the green predictive area, which means the frost-damaged shotcrete specimens with different geometries are precisely predicted. The calculated methods of fracture toughness of damaged shotcrete are verified to be suitable for specimens with various geometries.

### 4.3. The Influence of Different Initial Crack Length

**Figure 6:**
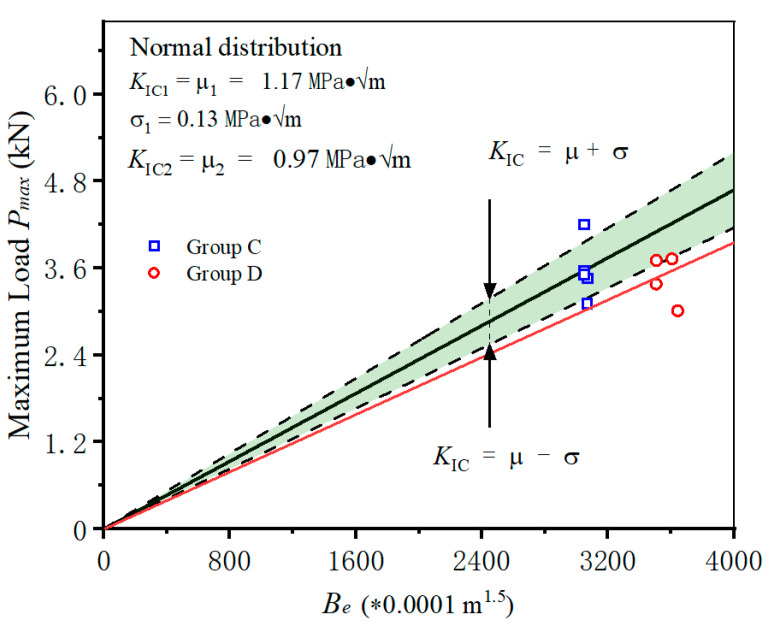
The fracture results in Group D are out of the range of the Group C predictive area.

**Figure 7:**
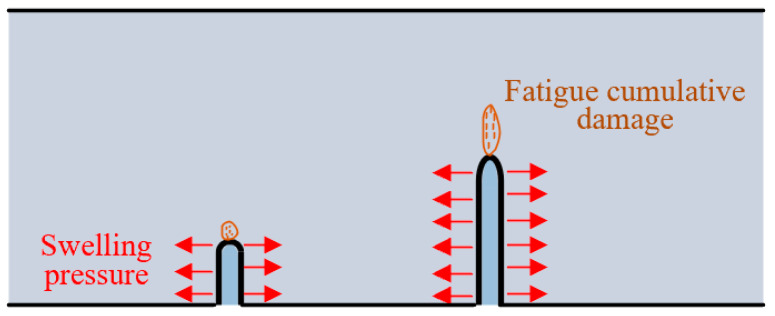
Comparing the fatigue cumulative damage of the crack tip between notches of different initial length.

In this section, we describe how the specimens with the same width but different initial crack lengths (Group C and Group D) were used to investigate the influence of initial crack length on fracture of frost-damaged shotcrete. The fracture toughness value of each specimen in Group C is first calculated, and then its distribution is analyzed through normal distribution analysis. As illustrated in Figure 6, the triangle predictive area of group C is established; the data from Group D are also plotted in the same coordinates. It can be found that a noticeable decline in fracture toughness (from 1.17 to 0.97 MPa·√m) occurs with the increase in initial crack length. When the saturated specimens suffer freeze–thaw cycles, the water in the initial crack will freeze and swell; the swelling pressure will be applied along the notch surfaces, as shown in Figure 7. With the increase in initial crack length, the frost-heaving force will be increased accordingly [37,38]. After several freeze–thaw cycles, the crack tip of shotcrete specimens with longer initial crack length will accumulate more damage. Consequently, the failure loads will decrease, and the calculated fracture toughness will be reduced. Therefore, when applying the calculation methods in Section 3 to assess the fracture toughness of cracked shotcrete structures in cold region, it is essential to measure the real crack length.

### 4.4. The Influence of Freeze–Thaw Cycles

**Figure 8:**
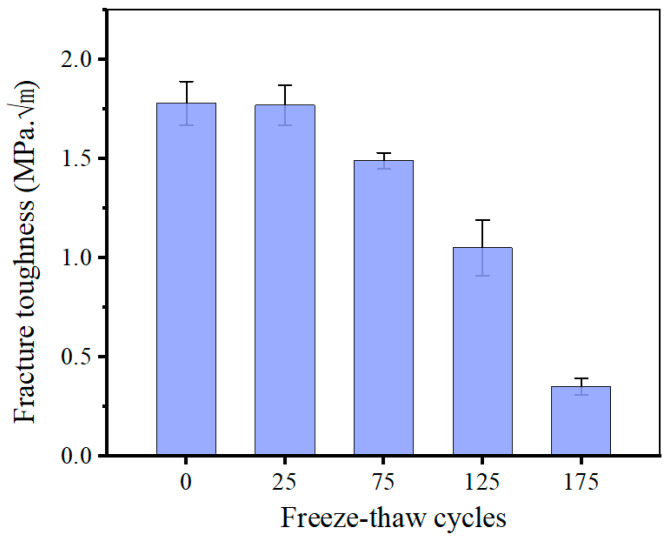
The variation in the fracture toughness of shotcrete specimens with different freeze–thaw cycles.

Typically, shotcrete in cold regions will repeatedly undergo freeze–thaw cycles throughout its service life. In this study, a total of 0, 25, 75, 125, and 175 freeze–thaw cycle experiments were conducted on shotcrete specimens, respectively. The maximum failure loads of each specimen could be obtained from 3-p-b fracture tests. Then, by substituting the maximum failure loads into Equation (5), the fracture toughness values are determined accordingly. As shown in Figure 8, the average fracture toughness values in five groups are 1.78, 1.77, 1.49, 1.05, 0.35 MPa·$\sqrt{m}$, respectively. During the initial 25 freeze–thaw cycles, the damage can be neglected, and the fracture toughness remains constant. When the freeze–thaw cycle reaches 75, a significant decline is witnessed for the fracture toughness of shotcrete specimens, which is caused by repetitive frost damage. When the shotcrete specimens have suffered 175 cycles of freeze–thaw, the fracture toughness value is only 20% of the original strength; the shotcrete materials almost lost their ability to withstand normal loads. These phenomena have already been proved in several published studies [39,40], which means that the established methods can be used to determine the fracture parameters of shotcrete specimens under various freeze–thaw cycles.

### 4.5. The Influence of Internal Damage

Fracture is one of the most common failure modes of shotcrete materials, and freeze–thaw damage makes the fracture process more complex. Hence, fracture analysis and the precise fracture prediction of freeze–thaw damaged shotcrete is crucial for its application in cold regions.

**Figure 9:**
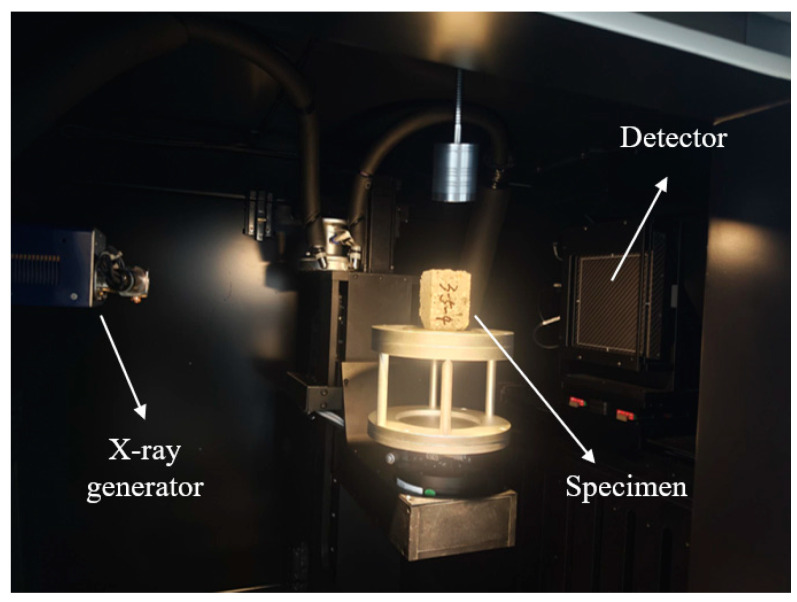
The illustration of computed tomography (CT) devices.

**Figure 10:**
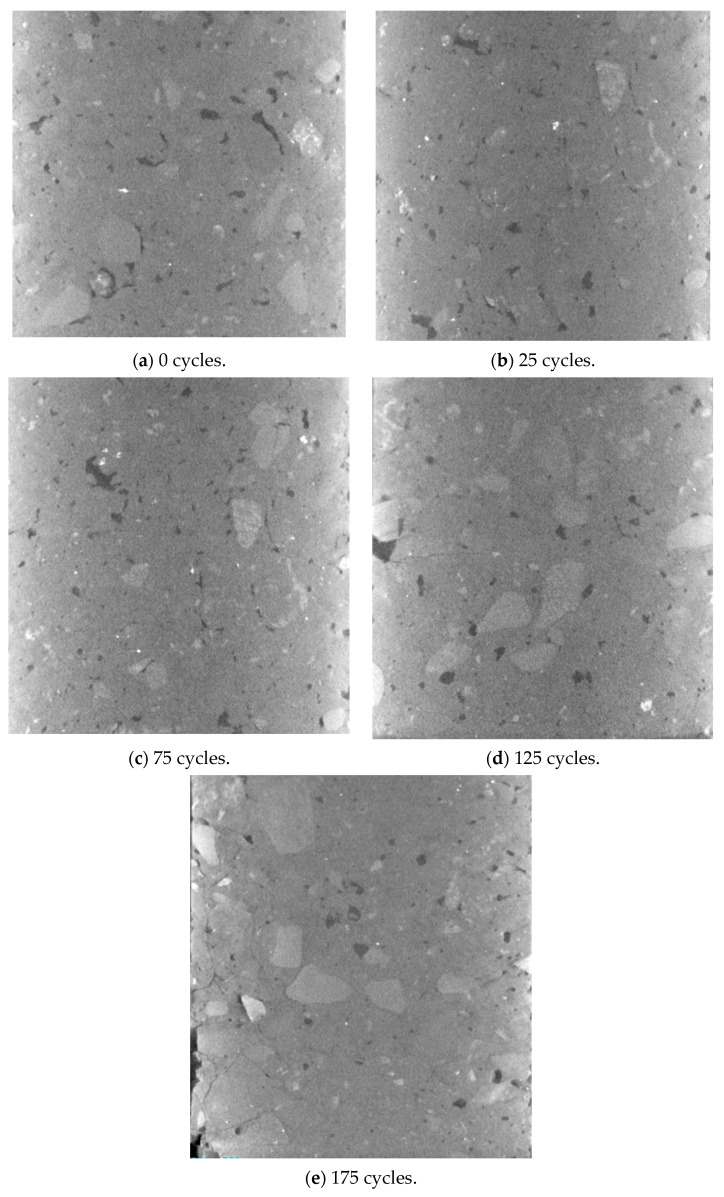
The CT scan images of shotcrete specimens after different freeze–thaw cycles.

Computed tomography (CT) scan devices are applied here to analyze the damage caused by freeze–thaw cycles to shotcrete materials from a microstructural point of view. As shown in Figure 9, the specimen is placed on a pallet. As the X-rays pass through the specimens, they are attenuated differently by shotcrete materials according to the density distribution. Consequently, the internal damage of shotcrete specimens can be acquired by tomographic reconstruction, which produces a series of cross-sectional images. The CT images of shotcrete specimens with different cycles of freeze–thaw are collected and compared, as shown in Figure 10. As observed, shotcrete materials inherently possess defects even before undergoing freeze–thaw cycles. Once the freeze–thaw cycle reaches 25, the variation in internal defects becomes negligible, which aligns with the fracture test results from Groups A1 and A2. However, as the number of freeze–thaw cycles increases, both the quantity and size of internal defects grow significantly. Initially, cracks appeared at the specimen’s periphery and, with successive freeze–thaw cycles, gradually extended toward the center. Over time, these cracks merged, leading to an increase in crack size. Furthermore, most of the cracks were located around the aggregates, which mirrors the fracture path observed in specimens without freeze–thaw damage. This progression explains the notable reduction in fracture toughness after a certain number of freeze–thaw cycles. It can also be concluded that the fracture performance of shotcrete is closely linked to its microstructural damage.

## 5. Conclusions

Shotcrete structures always suffer frost damage in cold regions, leading to more complex fractures. In this study, shotcrete specimens were cast in the laboratory, and 3-p-b fracture tests were subsequently conducted on shotcrete specimens after freeze–thaw cycles. Calculation methods for determining the fracture toughness of frost-damaged shotcrete were proposed, and several factors were studied to reveal the influence on fracture of frost-damaged shotcrete. The following conclusions can be drawn:

(1) The linear model was proposed to establish the relation between fracture toughness and the failure loads of frost-damaged shotcrete 3-p-b tests, which take into account not only specimen size, geometries, and notch length, but also the effects of specimen boundaries and microstructure. The distribution characteristics of fracture toughness were also analyzed and utilized.
(2) The influence of specimen geometries on the calculated fracture toughness can be ignored, and the obtained fracture toughness can be considered a material constant. However, the initial crack length cannot be ignored in the fracture toughness calculations. The longer the crack, the greater the damage near the crack tip during the freeze–thaw process, resulting in a lower calculated fracture toughness, which should be carefully considered in practical engineering assessments.
(3) Freeze–thaw cycles could lead to the formation of microstructural defects in shotcrete, with an evident impact on its resistance to fracture. In the initial 25 freeze–thaw cycles, the damage to the shotcrete is essentially negligible. As the number of freeze–thaw cycles increased, the microstructures of shotcrete were gradually damaged, the fracture toughness also deteriorated accordingly.

For future research, more emphasis will be placed on both field tests and laboratory experiments that replicate the real freeze–thaw conditions experienced by shotcrete. This approach will allow for a more comprehensive understanding of its performance, and the reliability of the established model can be further validated.

## Figures and Tables

**Table 1:** Chemical and physical properties of the superplasticizer.

Water-Reducing Rate (%)	Solid Content (%)	Density (g/mL)	Time-Loss of Slump (mm)	Entrained Air Content (%)	Compressive Strength Ratio (%)	
					7 d	28 d
30	19.6	1.063	25	2.1	153	145

**Table 2:** Chemical and physical properties of the accelerator.

Major Components	Solid Content (%)	Density (g/mL)	Chloride Ion Content (%)	Alkali Content (%)	Setting Time (s)	
					Initial Setting	Final Setting
Aluminum sulfate	59.5	1.406	0.017	0.05	166	390

**Table 3:** The testing conditions of specimens in different groups.

Group	Span (mm)	Height (mm)	Thickness (mm)	Initial Crack Length (mm)	Freeze–Thaw Cycles	Average Failure Load (kN)	Number
A	150	50	50	10	100	2.30	6
B	200	50	50	10	100	1.66	6
C	200	80	50	15	100	3.57	6
D	250	100	50	20	100	3.45	6
E	125	50	50	10	0	3.78	6
F					25	3.90	6
G					75	3.30	6
H					125	2.35	6
I					175	0.79	6

**Table 4:** The calculated fracture toughness value of each damaged shotcrete specimen.

No.	*P*_max_ (kN)	*K_IC_* $(\mathrm{MPa}\cdot \sqrt{m}$)	No.	*P*_max_ (kN)	*K_IC_* $(\mathrm{MPa}\cdot \sqrt{m}$)	No.	*P*_max_ (kN)	*K_IC_* $(\mathrm{MPa}\cdot \sqrt{m}$)
A1	1.78	1.02	D1	3.38	0.96	F6	3.86	1.82
A2	1.77	0.96	D2	3.01	0.83	G1	3.45	1.56
A3	2.70	1.58	D3	3.73	1.03	G2	3.12	1.48
A4	2.65	1.52	D4	3.71	1.06	G3	3.25	1.46
A5	2.61	1.49	E1	3.49	1.67	G4	3.20	1.44
B1	1.70	1.31	E2	3.88	1.89	G5	3.41	1.50
B2	1.50	1.16	E3	3.78	1.78	G6	3.42	1.51
B3	1.83	1.41	E4	3.96	1.85	H1	1.99	0.88
B4	1.56	1.20	E5	3.52	1.64	H2	2.66	1.13
B5	1.72	1.33	E6	4.02	1.87	H3	2.61	1.19
C1	3.46	1.13	F1	3.96	1.70	H4	2.15	0.98
C2	3.11	1.01	F2	4.25	1.90	I1	0.88	0.38
C3	4.20	1.38	F3	4.18	1.84	I2	0.69	0.30
C4	3.56	1.17	F4	3.50	1.64	I3	0.83	0.38
C5	3.51	1.15	F5	3.66	1.72	I4	0.76	0.34

## Data Availability

The original contributions presented in this study are included in the article. Further inquiries can be directed to the corresponding authors.
